# Scalable fabrication of microneedle arrays via spatially controlled UV exposure

**DOI:** 10.1038/micronano.2016.49

**Published:** 2016-10-10

**Authors:** Hidetoshi Takahashi, Yun Jung Heo, Nobuchika Arakawa, Tesuo Kan, Kiyoshi Matsumoto, Ryuji Kawano, Isao Shimoyama

**Affiliations:** 1 Department of Mechano-Informatics, Graduate School of Information Science and Technology, the University of Tokyo, 7-3-1 Hongo, Bunkyo-ku, Tokyo 113-8656, Japan; 2 Department of Mechanical Systems Engineering, Graduate School of Engineering, Tokyo University of Agriculture and Technology, 2-24-16 Naka-cho, Koganei-shi, Tokyo 184-8588, Japan; 3 Department of Mechanical Engineering and Intelligent Systems, Graduate School of Informatics and Engineering, the University of Electro-Communications, 1-5-1 Chofugaoka, Chofu-shi, Tokyo 182-8585, Japan; 4 IRT Research Initiative, the University of Tokyo, 7-3-1 Hongo, Bunkyo-ku, Tokyo 113-8656, Japan; 5 Department of Biotechnology and Life Science, Graduate School of Engineering, Tokyo University of Agriculture and Technology, 2-24-16 Naka-cho, Koganei-shi, Tokyo 184-8588, Japan

**Keywords:** inclined/rotated lithography, microneedle array, UV exposure ratio

## Abstract

This paper describes a theoretical estimation of the geometry of negative epoxy-resist microneedles prepared via inclined/rotated ultraviolet (UV) lithography based on spatially controlled UV exposure doses. In comparison with other methods based on UV lithography, the present method can create microneedle structures with high scalability. When negative photoresist is exposed to inclined/rotated UV through circular mask patterns, a three-dimensional, needle-shaped distribution of the exposure dose forms in the irradiated region. Controlling the inclination angles and the exposure dose modifies the photo-polymerized portion of the photoresist, thus allowing the variation of the heights and contours of microneedles formed by using the same mask patterns. In an experimental study, the dimensions of the fabricated needles agreed well with the theoretical predictions for varying inclination angles and exposure doses. These results demonstrate that our theoretical approach can provide a simple route for fabricating microneedles with on-demand geometry. The fabricated microneedles can be used as solid microneedles or as a mold master for dissolving microneedles, thus simplifying the microneedle fabrication process. We envision that this method can improve fabrication accuracy and reduce fabrication cost and time, thereby facilitating the practical applications of microneedle-based drug delivery technology.

## Introduction

Microneedles have been explored as a new class of effective transdermal drug delivery systems (DDSs) that offer minimally invasive, less painful, and self-administrable delivery^1–4^. Whereas conventional microfabrication techniques can produce only 2-dimensional or 2.5-dimensional microstructures, microneedle fabrication requires non-conventional microfabrication techniques that can achieve a cone-like, 3-dimensional (3D) geometry with a high aspect ratio and a sharp tip. Moreover, provided that the fabrication method is scalable, such a method could offer a means of achieving best-fit geometries for applications ranging from cosmetics to vaccinations. Because factors such as fabrication accuracy and cost are crucial for the use of microneedle-based DDS technology in practical applications, it is necessary to simplify fabrication to decrease fabrication cost and time while achieving sufficient fabrication accuracy.

To simplify the fabrication process of microneedle arrays, various micromanufacturing methods, including drawing lithography^5–7^, deep X-ray lithography of Lithographie Galvanoformung Abformung (LIGA)^8,9^, and ultraviolet (UV) lithography^10–15^, have been studied. Compared with other methods, UV lithography provides a simple, easily accessible, and conventional fabrication process for the creation of wide-area microneedle arrays. Among UV lithography-based methods, controlling the spatial distribution of the UV exposure of the photoresist enables the creation of microneedles via a single UV exposure step and a single development process. Hence, the complexity of the fabrication process can be significantly reduced. Previously, fabrication using integrated lens has been proposed for the creation of microneedle structures^10–13^. This integrated lens-based fabrication technique produces a large array of microneedles in a simple manner, but the technique is not scalable; modifying the microneedle geometry requires an entirely separate fabrication process to be conducted to change the lens geometry.

**Figure 1:**
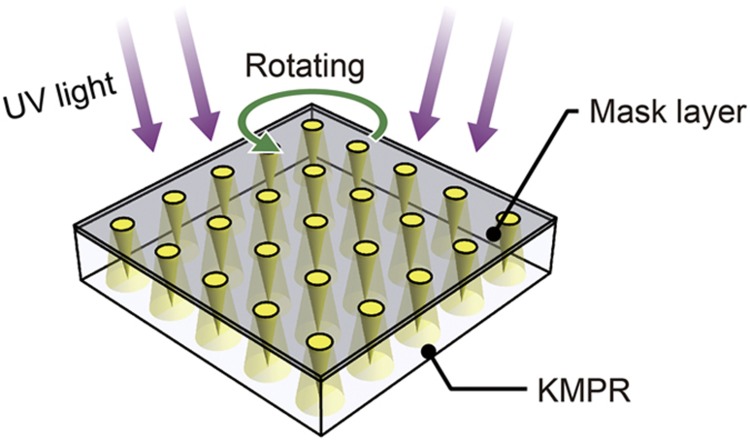
Schematic illustration of the fabrication of microneedle arrays via inclined/rotated UV lithography. UV, ultraviolet.

Among the available methods for the spatial control of UV exposure, inclined/rotated UV lithography enables the ideal scalable fabrication of microneedles, in which the photo-polymerized region can be modified without any additional fabrication steps. When inclined/rotated UV exposure is applied to a photoresist through circular mask patterns, microstructures with sharp-tipped conical shapes are obtained (Figure 1). In our procedure, in contrast to other approaches, the height of microneedles prepared using the same mask pattern can be controlled solely by controlling the inclination angle. At higher doses of UV exposure, the photo-polymerized region becomes larger, thus resulting in a larger volume of the microneedle body. Despite its potential for scalability, however, the fabrication of conical microneedle structures using inclined/rotated UV lithography has yet to be realized. In previous studies, inclined/rotated UV lithography has been used to obtain concave-shaped mold masters for microneedle fabrication (Supplementary Figure S1). This approach, compared with the direct fabrication of conical microneedle structures^11,16^, requires an additional molding step. Furthermore, no theoretical analysis of such inclined/rotated UV exposure has yet been performed, and this lack of a theoretical foundation hinders the understanding and use of inclined/rotated UV lithography for microneedle fabrication.

We propose a scalable method of fabricating microneedle structures via inclined/rotated UV lithography. We also describe a theoretical approach to understanding the 3D distribution of the UV exposure dose and estimating the microstructure geometry. When inclined/rotated UV exposure through circular mask patterns generates a conical-shaped distribution of the UV exposure dose in a negative photoresist, the photo-polymerized regions form microneedle arrays (Figure 1). As previously mentioned, by controlling the inclination angle and exposure dose, the photo-polymerized regions that are produced using the same mask patterns can easily be modified, thus allowing the facile development of microneedle structures with on-demand geometry. In this paper, we describe the theoretical analysis of the 3D distribution of the UV exposure dose in thick negative photoresist under inclined/rotated UV exposure. We also report the fabrication of microneedle arrays with varying inclination angles of the light source and varying UV exposure doses to demonstrate the accuracy of the theoretical estimation and the scalability of the proposed fabrication method.

## Materials and methods

### Materials

Negative photoresist was purchased from MicroChem (KMPR-1000 series; product number: KMPR 1035; Newton, MA, USA). Acetone (99.5%) and isopropyl alcohol (99.7%) were purchased from Wako Chemicals (Tokyo, Japan). Aluminum etchant was obtained by mixing H_2_PO_4_, HNO_3_, CH_3_COOH, and deionized water. H_2_PO_4_, HNO_3_, and CH_3_COOH were purchased from Wako Chemicals. Developer and positive photoresist were purchased from TOKYO OHKA KOGYO (product numbers: NMD-3 and OFPR-800LB 100cP; Kanagawa Prefecture, Japan). Cover glasses were purchased from Matsunami Glass (product number: Micro Cover Glass No. 4; dimensions: 0.35–0.45 mm in thickness, 40×30 mm; Chiba Prefecture, Japan).

### Microfabrication

**Figure 2:**
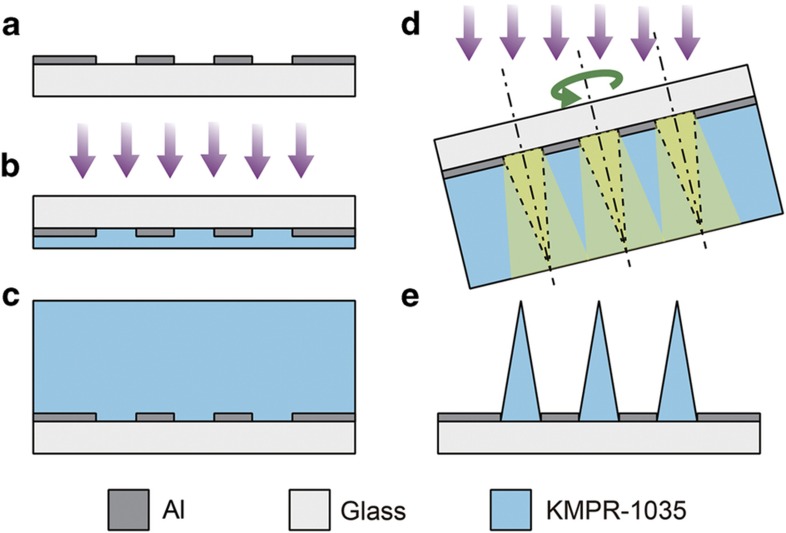
Process of fabricating microneedle arrays via inclined/rotated UV lithography. UV, ultraviolet.

We fabricated microneedle arrays from thick negative photoresist (KMPR 1035, MicroChem Corp.), using the inclined/rotated UV lithography technique to control the spatial distribution of the exposure ratio. We used KMPR, a widely used, thick, negative photopolymer. The process consisted of three main steps: depositing and patterning the masks on a glass substrate, irradiating the substrate with UV light through the mask pattern, and developing the exposed photoresist. First, an aluminum layer of 200 nm was deposited on a 40×30-mm glass substrate as a mask layer (Figure 2a). Then, circular patterns were etched in the aluminum on the substrate. Second, a KMPR layer of 50 μm in thickness was spin-coated onto the mask-pattern side of the substrate. This thin KMPR layer was exposed to UV light from the backside using a UV exposure apparatus (SUSS MA6/BA6 SPEC TU, SUSS MicroTec AG) (Figure 2b). The exposure dose was ~1100 mJ cm^−2^ in this exposure step. The light intensity was ~20 mW cm^−2^. The layer that was formed in this manner increased the adhesion between the glass substrate and the second layer of KMPR that was used to form the needle structures, thereby facilitating the adherence of the microneedles to the glass substrates after the developing process. Next, a 2-mm-thick KMPR layer was coated onto the first KMPR layer (Figure 2c). Then, we performed the main baking process, with a duration of ~4 h. This main baking process served as a post-exposure bake (PEB) for the first KMPR layer; such a PEB of sufficient length improves the adhesion between microneedles and a glass substrate^17,18^. After the main baking process, the photoresist layers were irradiated with inclined and rotated UV light from the backside of the glass substrate (Figure 2d). The substrate was fixed on a turntable with a rotation rate of ~3 r.p.m. (Supplementary Figure S2). The substrate was irradiated at exposure doses, *D*, of 1300, 1600, 1900, and 2200 mJ cm^−2^. The photoresist was developed in NMD-3 for 30 min after a 5-min PEB. After a hard bake process with a duration of 10 min, KMPR microneedle arrays were obtained on the glass substrate (Figure 2e). See Supplementary Information for further details of the fabrication process (Supplementary Figure S3).

## Theoretical calculations

We theoretically calculated the relative 3D distribution of the exposure ratio in the KMPR under inclined/rotated UV exposure on the basis of the relative exposure ratio for one rotation and the attenuation of UV light in KMPR, thus enabling the estimation of the needle shapes. The relative UV exposure ratio in an arbitrary *xy* plane determines the horizontal distribution of the exposure ratio. The attenuation of UV light in KMPR determines the vertical distribution. Diffraction has a role in determining the pattern resolution, particularly the radius of curvature of the microneedle tips. We discuss the effect of the diffraction of UV light in the subsection on UV light diffraction.

### Relative exposure ratio

**Figure 3:**
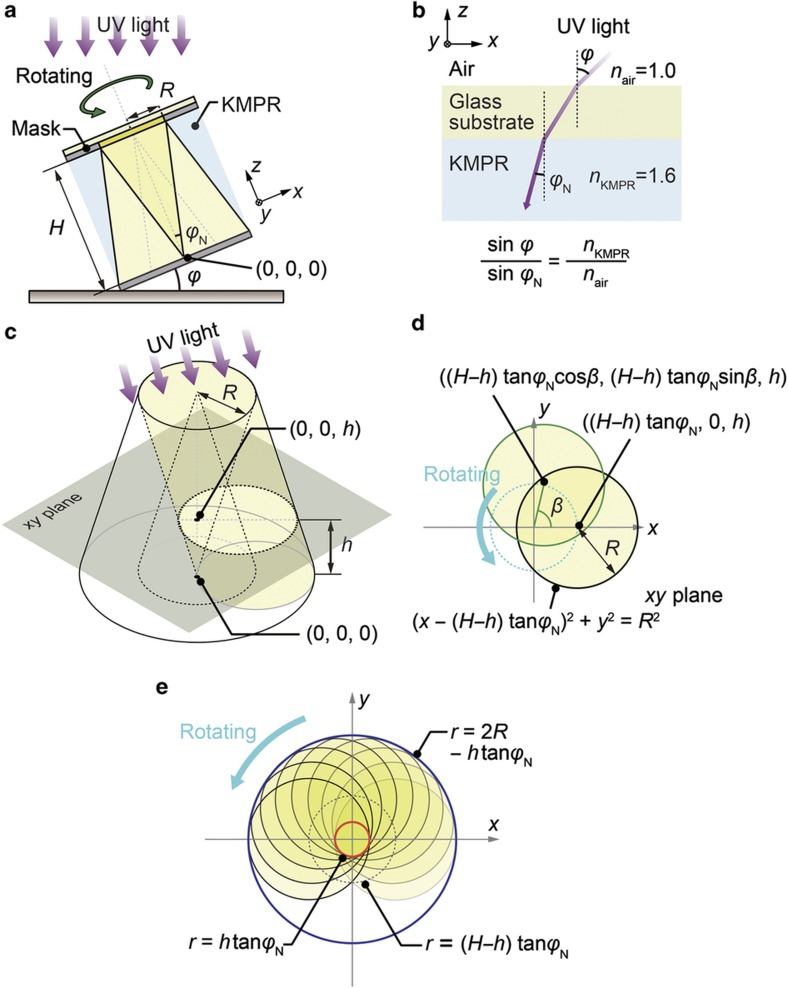
(**a**) Design parameters for microneedle fabrication: *R*, *φ*, *φ*_N_, and *H*. (**b**) Relationship between *φ* and *φ*_N_ according to Snell’s law. (**c**) Definition of the origin point and the *xy* plane at *z=h*. (**d**) Definition of the rotation angle *β* of UV exposure and the corresponding points in the *xy* plane shown in **c**. (**e**) The UV light trajectory and the boundary lines of the region that is exposed to UV light constantly (red circle) and the non-irradiated area (blue circle) UV, ultraviolet.

The main parameters that determine the exposure dose distribution are the radius of the mask pattern, *R*, and the inclination angle of the table, *φ*, as shown in Figure 3a. Consider a photoresist-coated substrate on a rotating inclined table that is exposed to UV light from the back side^19^. The relationship between the semi-vertex angle of the cone, *φ*_N_, and the inclination angle of the table, *φ*, satisfies Snell’s law^20,21^, as given below (Figure 3b):

(1)sinφsinφN=nKMPRnair

The refractive index, *n*, differs depending on the material; the refractive index of KMPR is 1.617. When *n* is smaller for the same inclination angle, the semi-vertex angle becomes larger and, consequently, the tip angle also increases. Thus, the refractive index affects the tip sharpness. Neglecting the effect of UV light attenuation in KMPR, the constantly UV-irradiated region has a conical shape. The height of the cone, *H*, is calculated from the radius of the mask pattern and the semi-vertex angle as shown below:

(2)H=RtanφN

We define the *xy* plane and the *z* axis as the plane parallel to the substrate and the axis vertical to the *xy* plane, respectively. We also define the vertex of the cone as the origin of the *xyz* axes, (0, 0, 0), and focus on the *xy* plane when *z=h*, as shown in Figure 3c.

Although the substrate experiences multiple rotations during inclined/rotated UV exposure, we assume only one rotation for this calculation. The rotation angle *β* varies from 0 to 2π radians, as shown in Figure 3d.

During one rotation, the relative exposure ratio at an arbitrary point can be calculated from the ratio between the irradiated and non-irradiated angles. For example, the relative exposure ratio is equal to 1 at points that are constantly irradiated throughout the entire rotation, whereas the relative exposure ratio is zero at points that are never irradiated throughout the entire rotation.

When *z*=*h*, the center of the irradiated circle is ($(H-h)\tan\varphi_{N}\cos\beta ,\;(H-h)\tan\varphi_{N}\sin\beta ,h$). If *β* is 0, the center is located at ($\left(H-h\right)\tan\varphi_{N},\;0,h$). Then, the UV-exposed points are described as below:

(3)(x−(H−h)tanφNcosβ)2+(y−(H−h)tanφNsinβ)2<R2

The constantly irradiated points (inside the red circle in Figure 3e) satisfy the equation given below:

(4)x2+y2<(htanφN)2

By contrast, the points that satisfy the equation given below (outside the blue circle) are never irradiated:

(5)x2+y2>(2R−htanφN)2

These equations can be expressed in polar coordinates <*r*, *θ*> as given below:

(6){r<htanφNr>2R−htanφN

Thus, the relative exposure ratio is determined solely by *r* and is independent of *θ*. Here, we define a parameter *t* as the distance from the center (*x=*0, *y=*0), which satisfies the equation given below:

(7)htanφN<t<2R−htanφN

**Figure 4:**
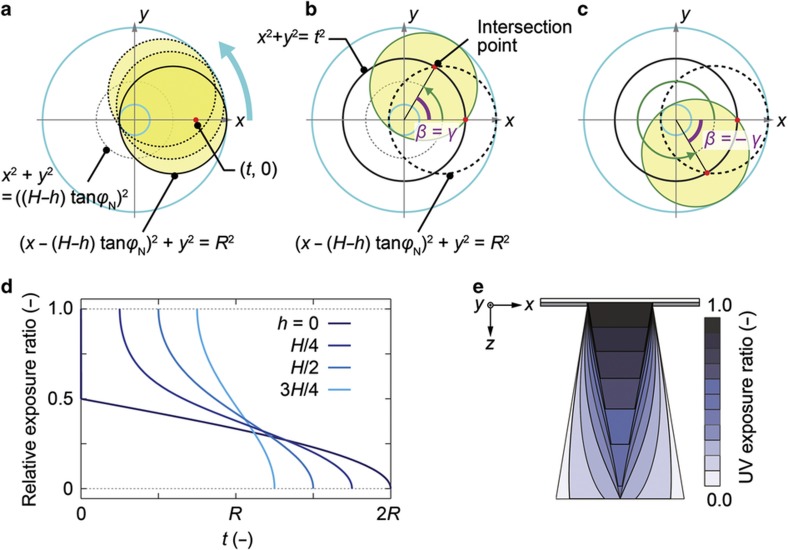
(**a**) Definition of the point <*t*, 0> in the *xy* plane and the rotating UV-exposed region. Yellow circles represent the UV-exposed region. In this calculation, the UV-exposed region is considered to rotate from 0 to 2π. (**b**) Definition of the angle *γ*. The point <*t*, *γ*> is located inside the UV-exposed region at the point of intersection between the black circle and the black dotted circle (*β=*0). (**c**) Definition of the angle 2π−*γ*. The point <*t*, 0> is again exposed to UV at rotation angles, *β*, of 2π−*γ* to 2π. Thus, the point <*t*, 0> is exposed to UV within the range of rotation angles from −*γ* to *γ*. The point <*t*, −*γ*> is located at the other intersection point between the black circle and the black dotted circle (*β=*0). (**d**) Relationships between the calculated relative exposure ratio and the distance *t* when *h*=0, *H*/4, *H*/2, and 3*H*/4. We define *t* as the distance from the center (*x*=0, *y*=0) of the occasionally irradiated area. (**e**) Calculated distribution of the UV exposure ratio in the *xz* plane when *y*=0. UV, ultraviolet.

The points that satisfy *r=t* are located in the occasionally irradiated region. In this region, the relative exposure ratio is between 0 and 1. We focus on the point <*t*, 0> (polar coordinate), which corresponds to (*t*, 0) (*xy* axis coordinate). Then, the UV-exposed area is rotated anticlockwise from a rotation angle of 0, as shown in Figure 4a. At a rotation angle of 0, the point <*t*, 0> is irradiated. The point <*t*, 0> is exposed to UV light until the rotation angle reaches *γ*, as shown in Figure 4b. The corresponding point <*t*, *γ*> within the irradiated region is located at the point of intersection between the black circle and the black dotted circle in Figure 4b, which are defined by the equations below:

(8){x2+y2=t2(x−(H−h)tanφN)2+y2=R2

Thus, the angle *γ* is determined by *t* and *h* and is calculated as shown below.

(9)γ=cos−1t2+((H−h)tanφN)2−R22(H−h)tanφN(0<γ<π)

When *β* becomes larger than *γ*, the point <*t*, 0> is no longer exposed to UV light. Similarly, UV light exposure resumes at a rotation angle of (2π−*γ*) and then persists through the end of the rotation, *β=*2π (Figure 4c). Therefore, the point <*t*, 0> is exposed to UV light for 2*γ* radians (−*γ*<*β<γ*) of one rotation. Thus, we can describe the relative exposure ratio as the ratio between the angle *γ* and the circular constant π.

When *h*=*H*, the relative exposure ratio is either 1 or 0 because all points are either constantly irradiated or constantly non-irradiated. Figure 4d shows the calculated relative exposure ratios as functions of *t* in the range of 0≤h<H. Because the relative exposure ratio is determined only by *t* and not by the rotation angle, the relative exposure ratio of a point at a distance *t* from the center line is described as given below:

(10)Relativeexposureratio=cos−1t2+((H−h)tanφN)2−R22(H−h)tanφN/π

As *h* decreases in the range of 0≤h<H, the occasionally irradiated area increases, as shown in Figure 4d.

### UV light attenuation

The attenuation of UV light is calculated from the Beer–Lambert law. The extinction coefficient *k* in KMPR at a wavelength of 365 nm (i-line) is ~4.0×10^−5^ μm^−1^ (http://www.microchem.com/Prod-KMPR.htm). Thus, the UV light intensity can be described as follows:

(11){I=I0e−α(H−hcosφN)α=4πkλ=1.38×10−3

*I*_0_ and *α* are the intensity at the substrate surface and the absorption coefficient, respectively. Thus, the UV light intensity ratio, *I*/*I*_0_, can be used to express the UV attenuation as a function of the height, *h*.

### UV exposure ratio

The theoretical exposure dose distribution can be estimated from the UV exposure ratio determined by the two aforementioned parameters. Regardless of the attenuation of UV light in the photoresist, the UV exposure ratio can be obtained from the relative exposure ratio in the *xy* plane at a given height *h* in *z*. By multiplying the relative exposure ratio by the UV light attenuation ratio at *h*, we can obtain the relative exposure ratio under nearly real conditions.

During inclined/rotated UV lithography, the UV light intensity at a point in the photoresist is given by the UV light intensity of the UV source multiplied by the UV exposure ratio at that point. Thus, we can estimate the shapes of the produced microneedles on the basis of the UV light intensity of the UV source and the UV exposure ratio distribution. We define the threshold level as the minimum UV light dose that can cure a portion of the photoresist, including the cone tip. For example, Figure 4e shows the distribution of the UV exposure ratio in the *xz* plane, where *y*=0 when the radius of the circular mask patterns *R*=100 μm and the inclination angle *φ*=15°, as calculated using Equations (10) and (11). Provided that the minimum UV light dose (threshold level) can cure the resist at a point where the UV exposure ratio is 0.4, we obtain a needle-shaped, photo-polymerized conical structure with a semi-vertex angle *φ*_N_ of 9.3°, a height *H* of 610 μm, and sharp tips, according to Equations (1) and (2). As the UV light dose from the UV source increases, the threshold level can cure a portion of the photoresist where the UV exposure ratio is <0.4, thus resulting in the expansion of the volume of the photo-polymerized region. Then, the tip angle of the cured portion becomes larger than the semi-vertex angle of the cone. As the UV light dose from the UV source decreases, the threshold level can cure the portion of the photoresist where the UV exposure ratio is >0.4, thus causing the volume of the photo-polymerized region to decrease. Such a decrease leads to imperfect needle structures with tip loss or rounded tip shapes. Therefore, we can estimate an appropriate UV exposure dose from the UV source that is needed to achieve a sufficient threshold level for the formation of microneedle structures for a given inclination angle and mask pattern radius.

### UV light diffraction

Previously, 3D structures of negative photoresist have been developed by utilizing diffraction effects^22–25^. Several studies have also described a physical model of exposure based on the Fresnel diffraction theory for inclined UV lithography^26–28^. In the fabrication of microneedle structures, diffraction is one of the factors that determine pattern resolution, particularly the radius of curvature of the microneedle tips. The diffraction effect can be ignored in the vicinity of the mask layer or at the bottom of the microneedles. For a fixed mask radius, the diffraction effect becomes stronger as the distance from the mask layer increases. Because the microneedle tips are formed at the far end of the microneedle structures and the tip sharpness of a microneedle is crucial for its skin penetration ability, the diffraction effect cannot be ignored at the microneedle tips, particularly in very tall structures.

The diffraction pattern produced by a circular aperture is known as the Airy diffraction pattern^29^. The radius of the central disk of the Airy diffraction pattern, *r*_d_, corresponds to the pattern resolution. The radius *r*_d_ at the microneedle tip can be calculated from the Fraunhofer diffraction equation (Supplementary Figure S4a) and is given as follows:

(12)rd=3.83⋅λ⋅H2π⋅R=3.83⋅λ2π⋅tanφN

where *λ* is the wavelength. The radius *r*_d_ is proportional to the height *H* and inversely proportional to the radius *R*. The radius *r*_d_ is also inversely proportional to tan*φ*_N_, as shown in Supplementary Figures S4b and c. Thus, the relative influence of diffraction increases as the semi-vertex angle of the cone, *φ*_N_, decreases. For example, if we define the tip resolution as 2*r*_d_ (Supplementary Figure S4a), then the tip resolution is 2.7 μm when *λ* is 365 nm and *φ*_N_ is 9.3°. The tip resolution reaches 10.2 μm when *φ*_N_ is 2.5°. Because a smaller *φ*_N_ leads to taller microneedles, the effect of diffraction on the tip resolution must be considered in the design of tall microneedles. By contrast, for smaller microneedles (*H*<1 mm), the diffraction effect on the tip resolution is relatively small. For example, when *R* is 100 μm and *H* is 540 μm, the experimental tip resolution is ~10 μm, whereas the tip resolution due to the diffraction is 2.7 μm.

## Results and discussion

**Figure 5:**
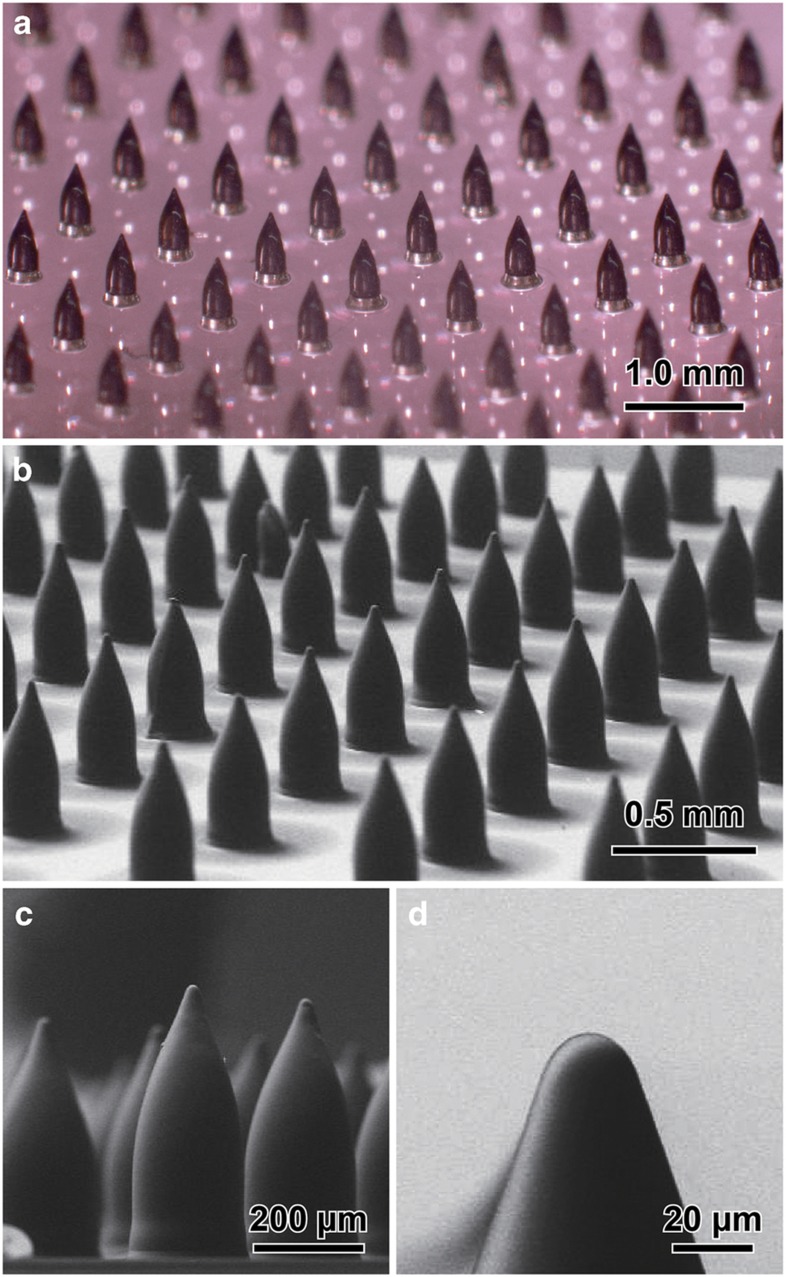
Microneedles fabricated using an inclination angle of 16° and a UV exposure dose of 1600 mJ cm^−2^. (**a**,**b**) A photograph and an SEM image of the fabricated device from a bird’s eye view. (**c**) An enlarged view of the SEM image in **b**. (**d**) An SEM image of a microneedle tip. The radius of curvature is 12 μm. SEM, scanning electron microscope; UV, ultraviolet.

We fabricated an array of 900 microneedles using a mask pattern radius, *R*, of 100 μm. The array was subdivided into 3-by-3 blocks, each containing a 10-by-10 microneedle array. Because we improved the adhesion between the KMPR and the glass substrates by means of a thin KMPR layer, all microneedles were attached to the glass substrate, even after the developing and rinsing steps. The pitch between the microneedles was 1200 μm. This pitch value was designed to avoid the overlap of any UV-exposed portions. We performed inclined/rotated UV lithography at an inclination angle of 16°. The inclination angle was determined from Equation (1) and the measured semi-vertex angle, *φ*_N_, of a microneedle that was fabricated without rotation (Supplementary Figure S5). Because the UV exposure intensities were uniform over a circular area of 4 inches in diameter, we were able to obtain an array of microneedles of uniform dimensions (Figures 5a and b). The microneedles showed a slightly asymmetric shape, primarily because of the contraction of the thick KMPR during the baking and cooling processes, mechanical stress during development, or an unstable rotation axis (Figure 5c). Although we were unable to achieve sharp microneedle tips identical to those indicated by the theoretical calculations, owing to the lithographic resolution of KMPR, the radius of curvature and tip angle were sufficiently small to penetrate skin layers (Figure 5d).

Microneedles fabricated in this manner could potentially be used for two purposes: as solid microneedles and as a mold master for dissolving microneedles. KMPR is a photoresist from the SU-8-negative epoxy series (MicroChem Corp.), which exhibit biocompatibility^30^, long-term stability *in vivo*^31^, sufficient mechanical strength^32^, and stability at body temperature to serve as solid microneedles. Because solid KMPR microneedles are sufficiently strong and sharp to penetrate rat skin, KMPR microneedles could penetrate the skin layers and leave residual holes in the skin (Supplementary Figure S6). If drugs were to be dispersed on the skin with residual holes, the drugs would diffuse through these residual holes. The other mode in which KMPR microneedles might be used is as a master for molding dissolving microneedles. If KMPR microneedle structures were to be used to cast polymer molds and these molds were then filled with biodegradable materials, dissolving microneedles could be obtained.

To verify the theoretical calculations of the microneedle geometry, we fabricated microneedles using varying UV exposure doses and inclination angles. We fabricated microneedles from a negative photoresist, KMPR, on cover glass substrates under four exposure doses *D* (1300, 1600, 1900, and 2200 mJ cm^−2^). At the same inclination angle of 16°, the microneedle geometry varied depending on the exposure dose.

**Figure 6:**
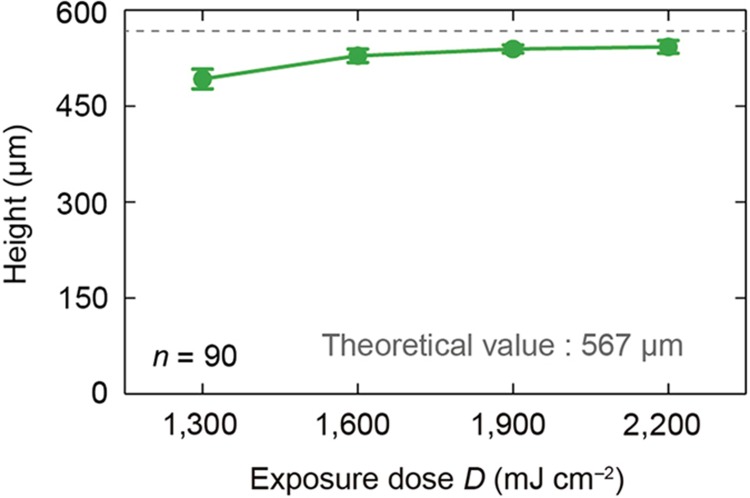
Experimentally obtained heights (*R*=100 μm) as a function of exposure dose. The theoretical height is 567 μm for a mask pattern radius of 100 μm.

The height of the microneedles formed at the UV exposure dose of 1300 mJ cm^−2^ was 490 μm, whereas the microneedle heights at doses of 1600, 1900, and 2200 mJ cm^−2^ were 530–540 μm. The average value for each exposure dose was calculated from scanning electron microscope (SEM) images of a 10-by-10 microneedle array from each block. In most cases, the heights were close to the theoretical microneedle height of 567 μm. However, because the minimum UV exposure dose in the cured region did not reach the threshold at the UV source exposure dose of 1300 mJ cm^−2^, we obtained microneedles of much smaller heights than the theoretical height (Figure 6).

**Figure 7:**
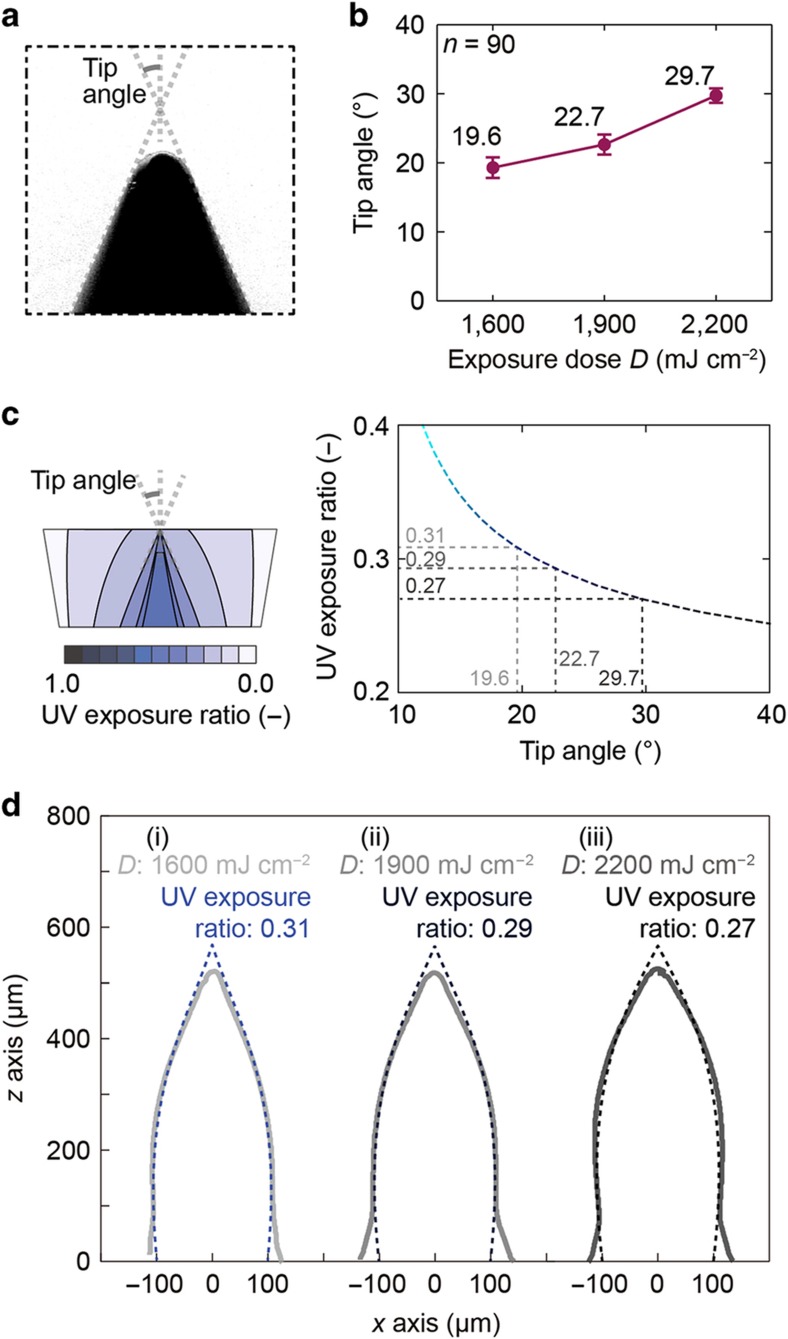
(**a**) Definition of the tip angle. (**b**) Tip angle as a function of exposure dose. (**c**) Theoretical relationship between the tip angle and the UV exposure ratio. We estimated the UV exposure ratio at the threshold level from the theoretical curve and the measured tip angle. A higher exposure dose can result in the curing of a photoresist region with a lower UV exposure ratio, thus resulting in a larger tip angle. (**d**) Comparison of the experimental and theoretical microneedle profiles due to the UV exposure dose distribution. UV, ultraviolet.

We also evaluated the radius of curvature and the tip angle of the microneedle tips for all UV exposure doses over the threshold (1600, 1900, and 2200 mJ cm^−2^). We estimated the tip angle from the tangent lines to a microneedle tip (Figure 7a). The radius of curvature (Supplementary Figure S7) and the tip angle (Figure 7b) increased as the UV exposure dose from the source increased. Thus, we were able to control the radius of curvature and the tip angle of microneedles fabricated in this manner by varying the exposure dose. The radii of curvature of the fabricated microneedles were <20 μm^33^. Although this dimension is considered to be sufficiently small to penetrate skin layers, the skin penetration capability of microneedles depends on not only the radius of curvature but also the shape and material of the needle body. For different materials, the inclination angle and UV exposure dose must be suitably optimized to fabricate skin-penetrating microneedles.

According to the theoretically calculated relationship between the tip angle and the UV exposure ratio, we estimated the UV exposure ratios at the threshold level from the measured tip angles (Figure 7c). Because the photoresist was cured above the threshold level, the threshold level determined the microneedle shape. The profiles of the fabricated microneedles matched well with the profiles of the UV exposure ratios at the threshold level (Figure 7d). However, discrepancies were observed at the bottoms and tips of the microneedles. Because the substrates were placed on a hot plate for the PEB step, the temperatures varied throughout the 3D microneedle structures. Thus, the temperature distribution during the PEB was the main cause of the observed discrepancies in the microneedle structures.

As shown in Figure 6, as the UV exposure dose from the light source was increased from 1300 to 2200 mJ cm^−2^, the microneedle height saturated at ~540 μm when the dose reached 1900 mJ cm^−2^; however, the microneedle volume continued to increase from 1900 to 2200 mJ cm^−2^ (Figure 7d). Thus, if the minimum UV exposure dose in the cured region is above the threshold level, the microneedle height does not increase as the UV exposure dose increases, but the microneedle volume does increase. The minimum UV exposure dose in the cured region can be calculated as

(13)calculatedUVexposureratio×UVsourceexposuredose×cosφN×transmittanceoftheglasssubstrate

The transmittance is defined as the ratio of the transmitted intensity to the incident intensity. The transmittance of the glass substrates was 0.93 for UV light passing through a single glass substrate at an inclination angle of 16°. Equation (13) describes the minimum UV exposure dose needed to cure the microneedles, as plotted in Figure 7. Together, the results indicate that the threshold level was ~500 mJ cm^−2^ (Supplementary Figure S8). Therefore, we were able to estimate the threshold for microneedle structures on demand. We also fabricated microneedle arrays with varying inclination angles. At the same UV exposure dose of 1900 mJ cm^−2^, as the inclination angle was increased, the height of the microneedles decreased (Supplementary Table S1). In addition, the microneedles formed using a small inclination angle and a large mask pattern radius and those formed using a large inclination angle and a small mask pattern radius did not form sharp tips, primarily because of insufficient exposure doses and over-threshold doses, respectively. In particular, in the case of the higher UV exposure dose, the cured region expanded outside of the microneedle tips, thus resulting in the formation of heart-shaped structures without sharp tips (Supplementary Figure S9a). These microneedles were fabricated with a mask pattern radius of 50 μm, an inclination angle of 22.5°, and a UV exposure dose of 1900 mJ cm^−2^. The result was similar to that indicated by the theoretical calculations, as shown in Supplementary Figure S9b.

We also calculated the sufficient UV exposure dose from the light source on the basis of the threshold level. The sufficient dose from the light source depends on the height of the microneedles because the UV exposure dose is attenuated from the bottom to the tip of a microneedle, such that the microneedle tip is cured at the lowest UV exposure dose. We define the sufficient UV exposure dose at the microneedle tip and the sufficient dose from the light source as *D*_tip_ and *D*, respectively. We then use Equation (11) to describe the relationship between *D*_tip_ and *D* as shown below:

(14)Dtip=DcosφNe−α(HcosφN)

The height is determined by the mask pattern radius and the inclination angle. Therefore, *D* is given as below:

(15)D∝eαRsinφNcosφN

The fabricated microneedles had a symmetric structure because of the circular nature of the mask patterns and the uniform rotational exposure. To demonstrate the potential for estimating the structure geometry realized through inclined/multidirectional UV lithography, we calculated the expected results for and performed inclined/two-directional UV lithography. The mask pattern radius was the same as in the experiment described above. The inclination angle was 45°, and UV light was emitted at two angles orthogonal to each other, as shown in Supplementary Figure S10a. Supplementary Figure S10b presents the UV exposure ratio. The ridge line of the structure can be calculated as shown below:

(16){x=uy=uz=u±1−u2tanφN

SEM images of the fabricated structures are shown in Supplementary Figures S10c and d. The UV source exposure dose was 360 mJ cm^−2^ at each angle. The structures were similar to ridges in the *yz* plane, and their height was ~300 μm. The geometry was similar to the theoretically estimated result with a UV exposure ratio threshold of 0.5. Therefore, our theoretical approach can be extended to the estimation of non-symmetric structures produced via inclined/multidirectional UV lithography.

As we experimentally demonstrated, inclined/rotated UV lithography provides a simple means of fabricating microneedles with on-demand geometries by controlling the UV exposure dose and inclination angle. For example, with an inclination angle of 7.5° and an exposure dose of 2200 mJ cm^−2^, we obtained microneedle arrays with a height of 900 μm (Supplementary Figure S11a). These microneedles could potentially reach the subdermal layer. Thus, such microneedle arrays may be suitable for delivering vaccines. By changing the inclination angle and exposure dose to 22.5° and 1000 mJ cm^−2^, respectively, we were able to decrease the height of the microneedles to 330 μm (Supplementary Figure S11b). Such microneedles could potentially reach depths below the epidermal layer but not below the deep dermal layers. These comparatively short microneedles are suitable for cosmetic applications. The aspect ratios (height/bottom diameter) of these microneedles are summarized in Supplementary Figure S12. Although the height for each inclination angle was slightly lower than the theoretical value because of fabrication errors (for example, errors in the inclination angle), the aspect ratios of the fabricated microneedles agreed well with the estimated values. Moreover, by increasing the exposure intensity, the body volume of the microneedles can be expanded, thereby enabling control of the drug content in microneedles of the same height. Such geometric control can be realized using the same mask patterns, and, thus, no additional fabrication process is required. This manner of achieving scalability by controlling the spatial distribution of UV exposure offers greater simplicity compared with previously developed microneedle fabrication techniques^10–13^. In addition, the present method can be used to fabricate microneedle arrays, whereas the previously developed inclined/rotated UV lithography method cannot directly produce microneedle shapes^11,16^. By virtue of this ability to directly fabricate microneedle arrays, the molding step can be eliminated, thereby simplifying the microneedle fabrication process.

Another advantage of the present work lies in the theoretical estimation of the microneedle geometry. We provide a means to calculate the 3D distribution of the UV exposure dose in a negative photoresist. From the calculated distribution of the UV exposure dose, the microneedle geometry can be estimated, including the maximum height, the tip angle, and the body profile. Because the estimated microneedle geometry agrees well with the fabricated microneedle geometry, our theoretical approach aids in understanding of inclined/rotated UV lithography, thus providing a simple route for microneedle fabrication under inclined/rotated UV exposure.

## Conclusion

This paper presents a theoretical analysis of inclined/rotated UV exposure and demonstrates the scalability of inclined/rotated UV lithography. The predictability and scalability of the proposed method may afford powerful advantages in the on-demand fabrication of microneedles for a wide variety of applications. In addition, we envision that this method can be applied for the fabrication of either solid microneedles or mold masters for dissolving microneedles, thereby simplifying the microneedle fabrication process. Therefore, the present method allows fabrication accuracy to be increased while reducing fabrication cost and time, thus facilitating the application of microneedle-based DDS technology for the development of practical transdermal DDSs.
