# Supplementary material for: Scalable fabrication of microneedle arrays via spatially controlled UV exposure
Source: Microsyst Nanoeng. 2016 Oct 10;2:16049. doi: 10.1038/micronano.2016.49 (PMC6444715; doi:10.1038/micronano.2016.49)
Supplement: Supplementary Information [file micronano201649-s1.pdf]

Supplementary file

Scalable fabrication of microneedle arrays via spatially controlled UV exposure

Hidetoshi Takahashi[1,*], Yun Jung Heo[2,*], Nobuchika Arakawa[2], Tesuo Kan[3], Kiyoshi Matsumoto[4], Ryuji Kawano[5] and Isao Shimoyama[1,4]

Microsystems & Nanoengineering (2016) **2,** 16049; doi:10.1038/micronano.2016.49; Published online: 10 October 2016

SUPPLEMENTARY MATERIALS AND METHODS

Microneedle insertion in rat skin

To demonstrate skin penetration ability, KMPR solid microneedles were inserted into cadaver rat skins. Cadaver rat skins were collected from two Sprague Dawley rats, which had been euthanized after they had been used for other research purposes. The subcutaneous fat was removed with surgery scissors. Hairs were completely removed using an electric pet clipper and hair removal cream. The skin samples were cleaned and stored at −30 °C until use. Microneedles were inserted by pressing on the backside of microneedle arrays with a thumb for ~2 min.

The arrays were removed and the area of insertion was stained with trypan blue. Trypan blue stained the sites of stratum corneum perforation. The excess stain was removed with paper tissues and the skins were washed with water.[1] The stained skins were viewed by a digital microscope (VHX 500, Keyence Corp., Osaka, Japan).

REFERENCE

1 Kochhar JS, Goh WJ, Chan SY, Kang L. A simple method of microneedle array fabrication for transdermal drug delivery. *Drug Development and Industrial Pharmacy* 2013; **39**: 299–309.

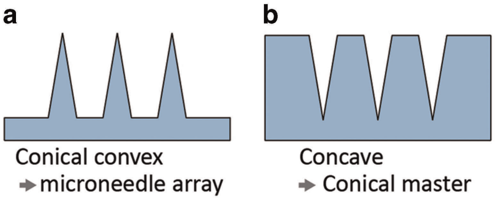

Figure S1 (**a**) Conical convex shape. (**b**) Concave shape.

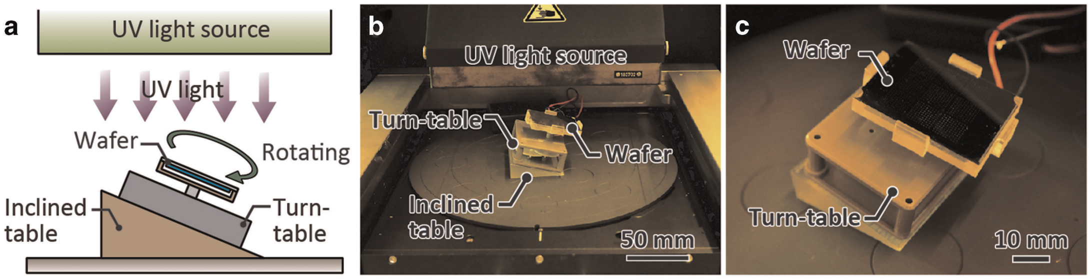

Figure S2 Experimental setup of the inclined/rotated UV lithography. (**a**) A photoresist coated wafer is placed on a turn-table; the turn-table is placed on an inclined table to achieve the inclined UV exposure. (**b**) Photo of the experimental setup described in **a**. (**c**) An enlarged view of the experimental setup in **b**.

[1]Department of Mechano-Informatics, Graduate School of Information Science and Technology, the University of Tokyo, 7-3-1 Hongo, Bunkyo-ku, Tokyo 113-8656, Japan; [2]Department of Mechanical Systems Engineering, Graduate School of Engineering, Tokyo University of Agriculture and Technology, 2-24-16 Naka-cho, Koganei-shi, Tokyo 184-8588, Japan; [3]Department of Mechanical Engineering and Intelligent Systems, Graduate School of Informatics and Engineering, the University of Electro-Communications, 1-5-1 Chofugaoka, Chofu-shi, Tokyo 182-8585, Japan; [4]IRT Research Initiative, the University of Tokyo, 7-3-1 Hongo, Bunkyo-ku, Tokyo 113-8656, Japan and [5]Department of Biotechnology and Life Science, Graduate School of Engineering, Tokyo University of Agriculture and Technology, 2-24-16 Naka-cho, Koganei-shi, Tokyo 184-8588, Japan.
Correspondence: Yun Jung Heo (yunjheo@cc.tuat.ac.jp) or Isao Shimoyama (isao@i.u-tokyo.ac.jp)
*These authors contributed equally to this work.

Cross sectional view	Process flow		Condition in detail
a 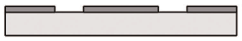	Wafer preparation	Glass wafer	40 mm × 30 mm
	Al depositing & pattering	Al deposition	Vacuum vapor deposition
		Prebake<br>Spincoat	Prebake 110°C 2 min<br>OFPR800-100cp<br>300 rpm 8 sec<br>2000 rpm 30 sec<br>6000 rpm 0.7 sec
		Mainbake	Main bake 110°C 2 min
		Exposure	Exposure 200 mJ cm^{-2}
		Developing	Develop NMD-3 20 min<br>(Rotate beaker 120 rpm)<br>1st water rinse<br>2nd water rinse
		Al etching	Al etchant 65 °C[a]<br>1st water rinse<br>2nd water rinse
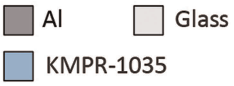		Cleaning	Acetone 5 min<br>IPA 2 min
b 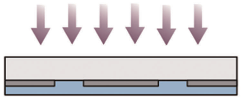	Coat a contact layer	Prebake	Prebake 160°C 5 min
		KMPR spincoat	KMPR-1035<br>Slope 5 sec<br>500 rpm 55 sec<br>Slope 5 sec<br>6000 rpm 55 sec
		Mainbake	Main bake 100°C 10 min
		Exposure from backside	Exposure 1100 mJ cm^{-2}
c d e 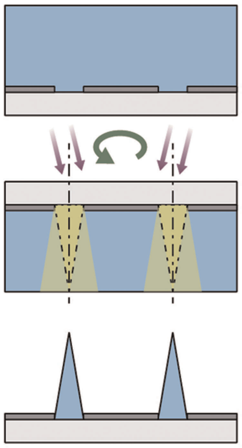	Fabrication of needle strusture	KMPR coat from backside	KMPR-1035<br>1.5 mm coat
		Mainbake	Mainbake 100°C 4 hours
		Inclined/rotated exposure from backside	Exposure x mJ cm^{-2}<br>(Rotational rate : 3 rpm)<br>(UV intensity :<br>20 mW cm^{-2})
		Post exposure bake	PEB 100°C 5 min
		Developing	Develop NMD-3 20 min<br>1st water rinse<br>2nd water rinse
		Post bake	Main bake 250°C 5 min

[a]$H_2PO_4:HNO_3:CH_3COOH:H_2O$ = 10:1:1:2

Figure S3 Process flow of the microneedle array fabrication in detail.

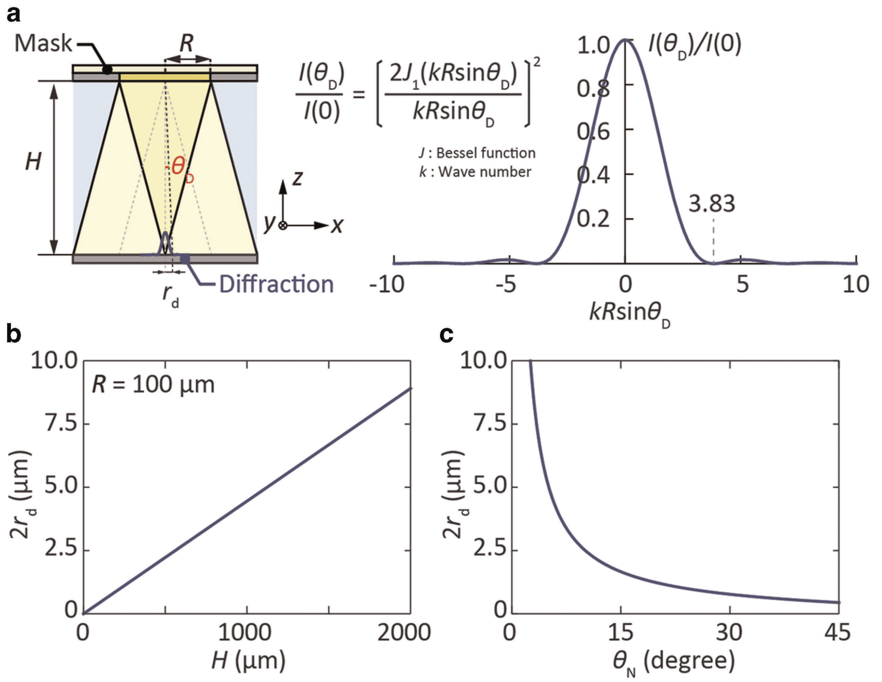

Figure S4 (**a**) Calculation of the influence of the UV light diffraction. (**b**) Relationship between height, H, and pattern resolution, $2r_d$. (**c**) Relationship between semi-vertex angle, φ_N, and pattern resolution $2r_d$.

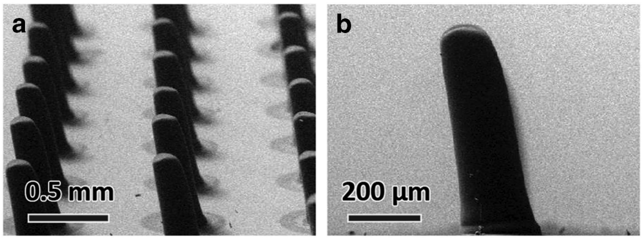

Figure S5 SEM images of the fabricated device without rotating.

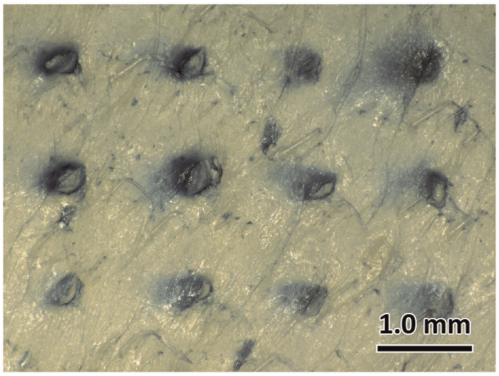

Figure S6 A photograph of penetration of skin layers with the fabricated microneedles with UV exposure dose of 1600 mJ cm^{-2}.

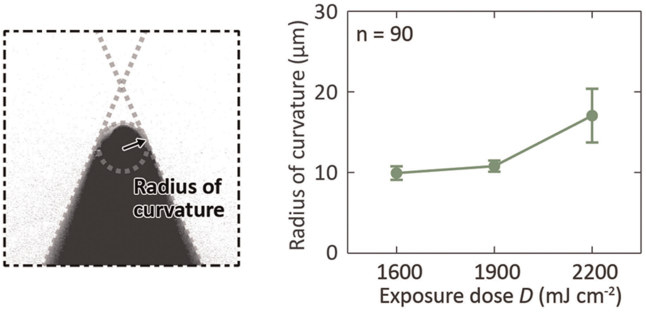

Figure S7 Relationship between exposure dose and radius of curvature.

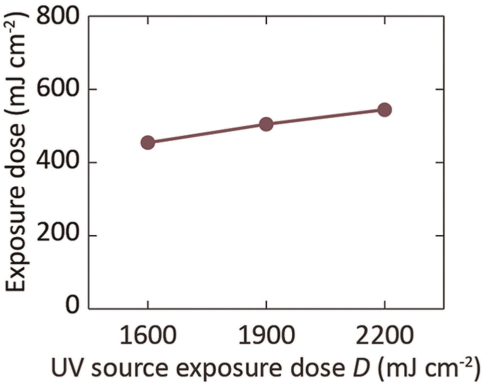

Figure S8 Relationship between exposure dose of the UV light source and the minimum exposure dose in the photo-polymerized portion. The minimum exposure dose can be estimated by multiplying the UV exposure dose and the UV exposure ratio at a threshold level.

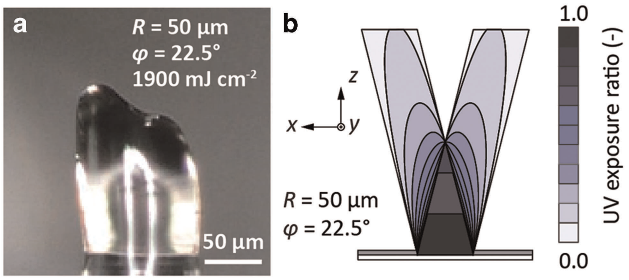

Figure S9 (**a**) Side view of the fabricated microneedle (R : 50 μm, φ : 22.5°). (**b**) Calculated distribution of the UV exposure ratio over the microneedle tip.

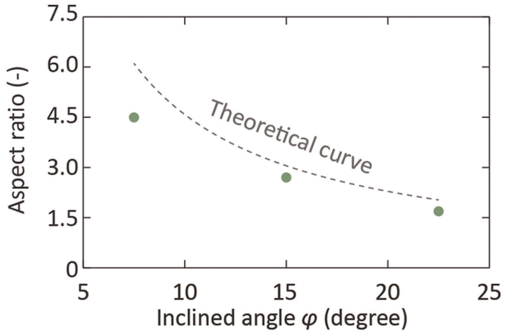

Figure S12 Relationship between the inclination angle and aspect ratio (height/bottom diameter) of the microneedles.

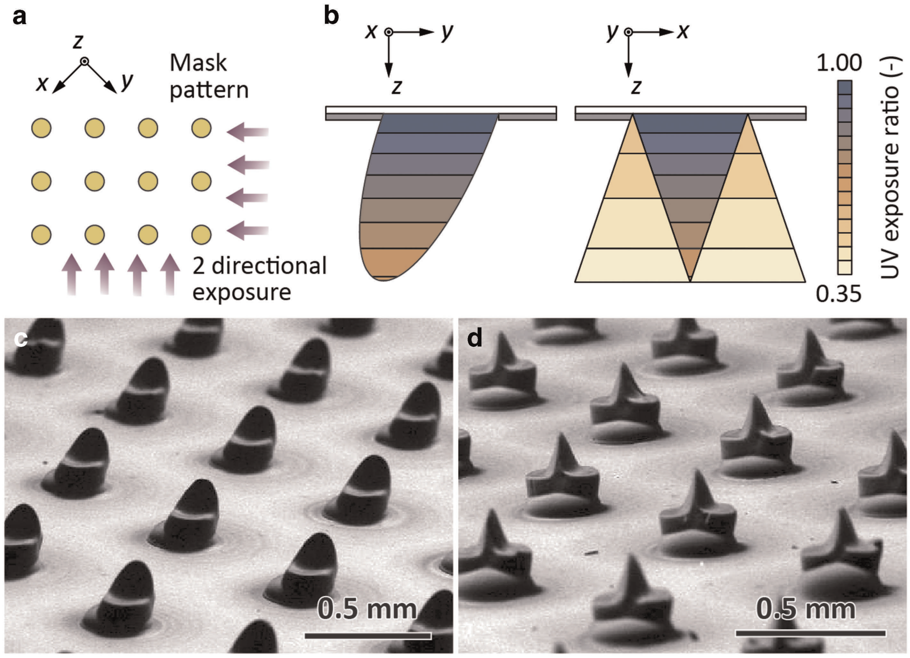

Figure S10 Demonstration of the fabrication of non-symmetric structures. (**a**) Conceptual illustration of two directional UV exposure. (**b**) Calculated distributions of the UV exposure ratio in the yz plane and xz plane. (**c**, **d**) SEM images of the fabricated structures.

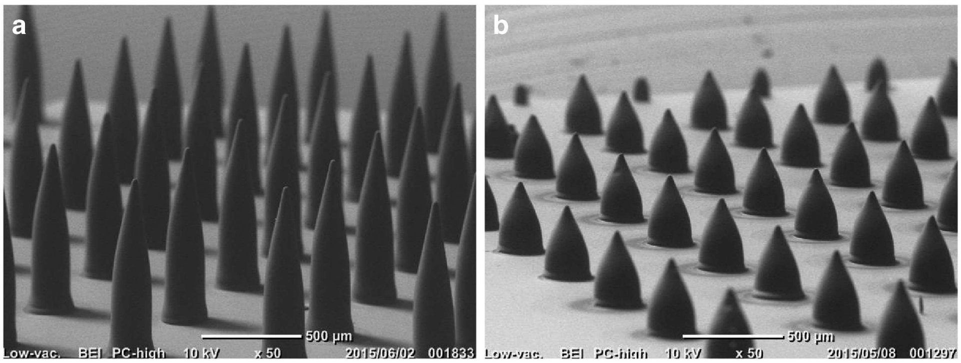

Figure S11 SEM images of the fabricated devices with inclination angles and exposure doses of (**a**) 7.5° and 2200 mJ cm^{-2} and (**b**) 22.5° and 1000 mJ cm^{-2}.

Table S1 Dimensions of microneedles for varying inclination angles of a table at a UV exposure dose of 1900 mJ cm^{-2}

Inclination angle of a table, φ (°)	Radius of mask pattern, R (μm)	Theoretical height, H (μm)	Diameter (bottom), $2R$ (μm)	Radius of curvature (tip) (μm)	Height, H (μm)
7.5	50	611	130 ± 4	19 ± 3	567 ± 13
	75	916	178 ± 2	32 ± 6	687 ± 27
	100	1222	228 ± 6	64 ± 1	760 ± 16
	125	1527	273 ± 2	107 ± 6	729 ± 3
15	50	305	107 ± 1	39 ± 3	318 ± 1
	75	458	155 ± 1	29 ± 1	436 ± 4
	100	610	208 ± 3	16 ± 0	560 ± 1
	125	763	258 ± 2	20 ± 1	644 ± 8
22.5	50	203	139 ± 2	–	262 ± 5
	75	304	191 ± 2	–	329 ± 2
	100	406	235 ± 3	104 ± 10	400 ± 4
	125	507	292 ± 3	64 ± 12	464 ± 6
